# Nanoscale Charge Density and Dynamics in Graphene Oxide

**DOI:** 10.1021/acsmaterialslett.1c00550

**Published:** 2021-11-22

**Authors:** Elisa Palacios-Lidón, Jaime Colchero, Miguel Ortuno, Eduardo Colom, Ana M. Benito, Wolfgang K. Maser, Andrés M. Somoza

**Affiliations:** † Departamento Física, Edificio CIOyN (Campus Espinardo), Universidad de Murcia, E-30100 Murcia, Spain; ‡ Instituto de Carboquímica (ICB-CSIC), E-500018 Zaragoza, Spain

## Abstract

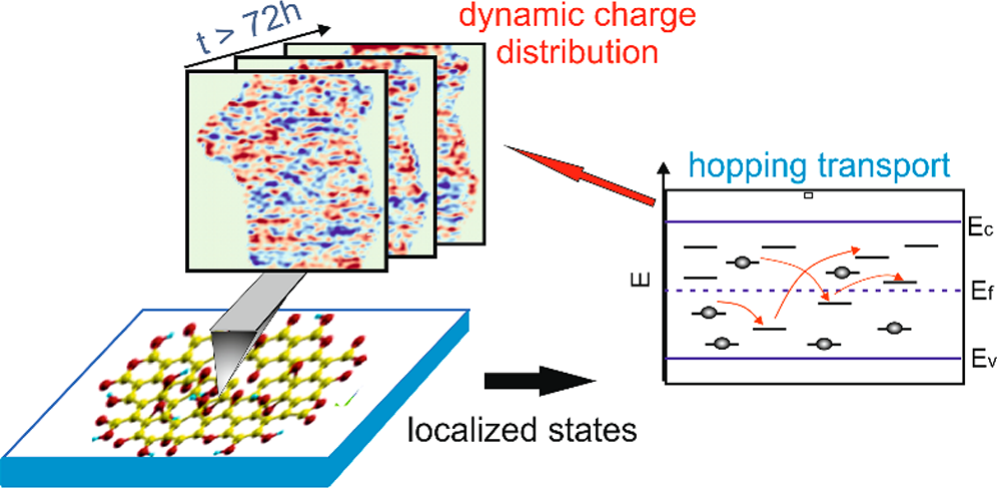

Graphene oxide (GO) is widely used as a component in thin film optoelectronic device structures for practical reasons because its electronic and optical properties can be controlled. Progress critically depends on elucidating the nanoscale electronic structure of GO. However, direct experimental access is challenging because of its disordered and nonconductive character. Here, we quantitatively mapped the nanoscopic charge distribution and charge dynamics of an individual GO sheet by using Kelvin probe force microscopy (KPFM). Charge domains are identified, presenting important charge interactions below distances of 20 nm. Charge dynamics with very long relaxation times of at least several hours and a logarithmic decay of the time correlation function are in excellent agreement with Monte Carlo simulations, revealing an universal hopping transport mechanism best described by Efros–Shklovskii’s law.

Graphene oxide (GO), a sheet of graphene decorated with various types of oxygen functional groups on its basal plane and edges, is a promising 2D carbon platform of special interest for thin film optoelectronic devices.^1−7^ Here GO offers unique advantages when it comes to gaining control on electrical and optical properties, accompanied by a highly favorable liquid-phase processing behavior. The random and inhomogeneous oxygen functional groups distribution confers GO a highly nonstoichiometric and disordered character and induces the formation of localized states within the bandgap.^8−10^ The oxygen atoms form sp^3^ bonds with carbon atoms in the basal plane, clustering in domains of several tens of nanometer in size,^11^ disrupting the extended sp^2^ conjugation network of an original graphene sheet generating also morphological defects. Its atomic structure can be viewed as an undulated semiamorphous solid carbon oxide, responsible for its electrically insulating behavior. Chemical and thermal reduction treatments aim on the removal of oxygen functional groups partially restoring the original sp^2^ network. The resulting reduced GO (rGO) with progressively increased sp^2^/sp^3^ ratios exhibits enhanced conductivities covering electrical transport regimes from variable range hopping (VRH) to semiconductor and semimetal regimes.^9,12−21^ Today, the charge transport behavior for rGO, being partially or progressively reduced, is quite established. However, there is a critical lack of corresponding knowledge in what concerns parent GO in its as-produced and highly oxidized form. Direct experimental access to the electronic structure, which is challenging because of its intrinsically nonconductive character,^19,22,23^ is yet needed to fully comprehend the reduction process of GO right from the very beginning. Understanding this would enable improved comparison with theoretical models and contribute to critical knowledge on the operational functionality of GO in optoelectronic devices.

A suitable technique for obtaining insight into the nanoscale charge transport properties is Kelvin probe force microscopy (KPFM). KPFM allows monitoring localized charges in low conducting systems.^24−27^ In the presence of localized charges, the KPFM signal (*V*_KPFM_) includes not only the contribution of the contact potential (*V*_CP_) but also the localized charge contribution (*V*_charge_).^24,25,28^ Recently, it has been shown that for 2D systems the charge distribution can be directly obtained from the KPFM images.^29,30^ This procedure is especially suited for GO where complex charge distributions are expected.

In this work, we have studied the nanoscale charge distribution and charge dynamics of individual GO flakes deposited from ultradiluted aqueous GO solutions on silicon substrates with a 300 nm SiO_2_ thick layer by means of KPFM measurements at room temperature. Using a mask-based analysis,^31^ we study the GO flakes and the SiO_2_ substrate separately, serving the latter as the reference for the *V*_KPFM_ measurements and to estimate experimental noise (σ_noise_). The acquisition parameters and the low relative permittivity of the underlying substrate has been carefully selected to optimize the *V*_charge_ signal. In addition, all the measurements have been carried out under N_2_ dry atmosphere to minimize physisorbed water screening effects (see SI.1 for further experimental details).

Topography and KPFM images (Figure 1A,B) show that for such low coverage, individual monolayer GO flakes are distinguished. The KPFM images reveal potential domains of several hundred of nanometers in size not related with the topography of the GO flakes (Figure 1B,D). In addition, the mean *V*_KPFM_ over a GO flake is about 80 mV with respect to the SiO_2_ substrate. This latter value is ascribed to the *V*_CP_ of the GO flake,^22^ while the potential domains are related with the V_charge_ contribution due to GO’s disordered insulating nature. It is known that the localization length (ξ) of the charges in GO and r-GO ranges from 0.5 to 4 nm depending on the sp^2^/sp^3^ fraction.^1,5^ Therefore, the observed *V*_charge_ domains should include the contribution of multiple charges. At *T* = 25 °C, we estimate from the Coulomb energy *E*_C_ = e^2^/4πε_0_κ*r*_0_ ≈ *kT* (κ = 4.3 for SiO_2_ and GO as explained in SI) a correlation charge distance of *r*_0_ ≈ 15 nm. This means, that at scales of *r* ≫ *r*_0_, the system behaves as a noninteracting one, which leads to large *V*_charge_ domains in accordance to simulations.^30^

**Figure 1:**
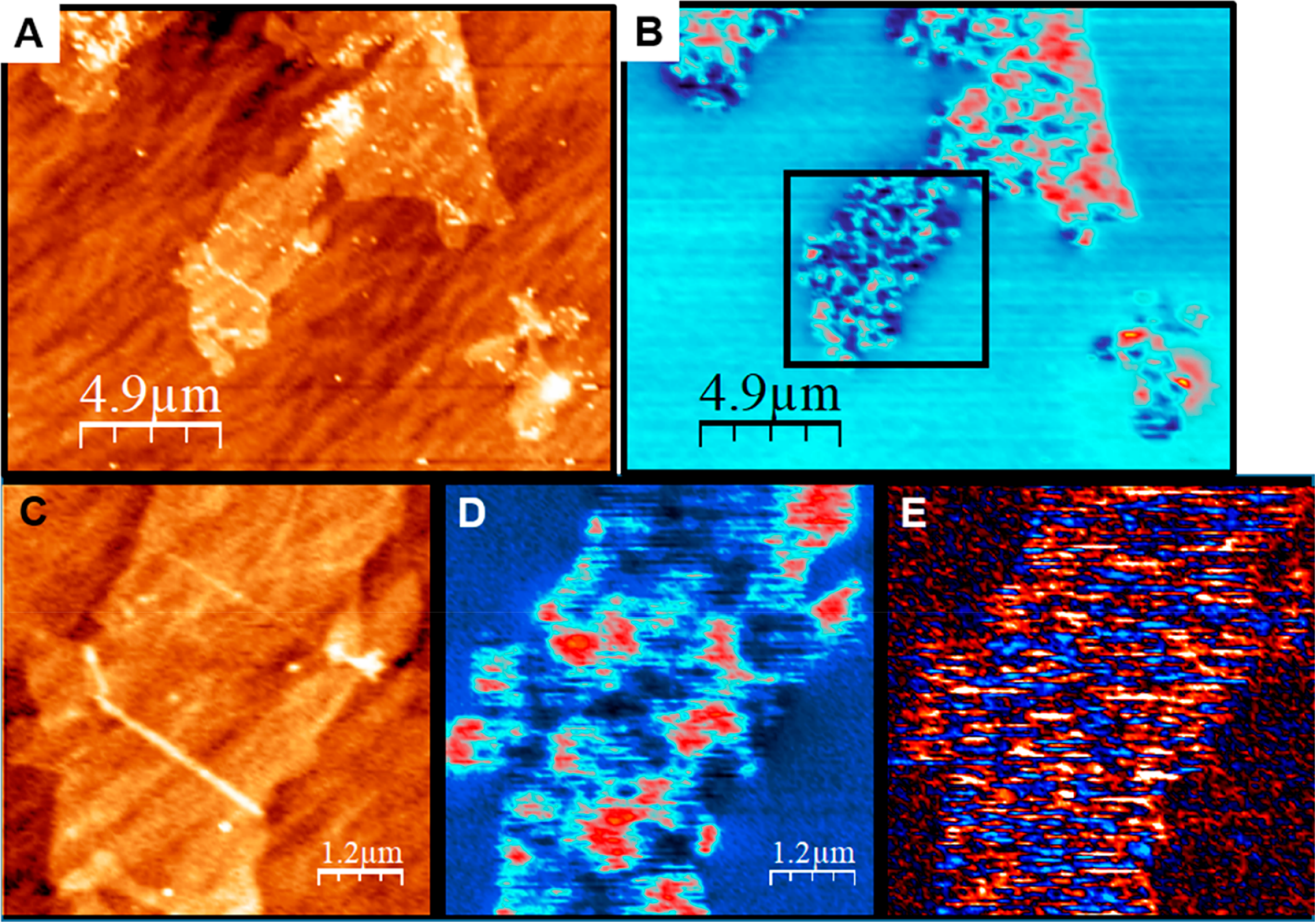
(A and C) Low- and high-magnification topography images of GO flakes and (B and D) corresponding KPFM images. (E) two pass subtracted image. The z-scale is (A and C) 4 nm, (B and D) 400 mV, and (E) ±50 mV.

Moreover, consecutive KPFM images show important dynamics ensuring that charges are hopping between localized states because of thermal fluctuations (SI Movie 1). A fingerprint of the charge dynamics in a disordered “hopping” system is the existence of a large range of time scales, from picoseconds to hours or even days.^32^ Considering the relatively slow KPFM image acquisition (typically 1–2 s/line in our experiments), we first analyze how charge movement may affect the data acquisition and interpretation. To do so, we use a two-pass method, where each line is scanned twice with exactly the same acquisition parameters. The direct subtraction of the two lines cancels all the contributions except those from the charges that have moved between measurements (Figure 1E and SI.2). On GO, we observe fluctuations that occur homogeneously all over the flake. Their amplitudes are not larger than ±60 mV but clearly exceed those observed over the SiO_2_ substrate (interpreted as the experimental noise level σ_noise_ ≈ 10 mV). We estimate that the average charge redistribution at an image point between the two passes (Δ*t* = 2 s) to produce such *V*_charge_ amplitude corresponds to a net charge movement roughly equivalent to one electron that travels a distance (*d*_l_) smaller than 10 nm (explained in the SI.3). We would like to emphasize that this travel distance is not the hopping distance of one single charge but rather the result of several charges hopping shorter distances.

To study the GO charge distribution and its dynamics, instead of using the *V*_KPFM_(*x*,*y*) images directly, we work with the corresponding charge density images (*q*(*x*,*y*)) obtained with the FFT deconvolution algorithms proposed in ref (29) (see SI.4 for further details on the deconvolution procedure). Because of experimental noise, this method requires signal filtering, which limits the lateral resolution, averaging the effects at a scale shorter than the filter cutoff radius (*R*_c_). Additionally, in order to determine the charge density at a point, the deconvolution assumes a static charge density in a surrounding area of radius *R*_c_. However, in a dynamic system, this requires that a charge should travel an effective distance (*d*) smaller than *R*_c_ during the scanning time (*t*_c_) of this area (*t*_c_ = *t*_l_*R*_c_*N*/*L*, with *t*_l_ being the line scanning time, *N* the number of images points, and *L* the image size). This introduces an additional constrain, as *R*_c_ should be larger than *d*. Assuming a charge diffusion process *d*^2^ ∝ *t*_c_, the above condition is fulfilled when,  where Δ*x* = *L*/*N* and *d*_l_ is the traveling distance in *t*_l_ of ∼10 nm, as previously estimated from our retrace experiments. In practice, to obtain a certain lateral resolution, it is necessary to reach a compromise between experimental noise, line scanning time and size and number of points of the image. In this situation, the method provides an accurate “coarse-grained” charge-density that properly describes the behavior of the system at scales larger than *R*_c_ and times larger than *t*_c_.

With this in mind, we will focus first on the overall behavior of the flake. A typical charge distribution obtained from a *V*_KPFM_ image is shown in Figure 2A. At this low magnification (*L*/*N* = Δ*x* = 54 nm, *t*_l_ = 2s, *R*_c_ = 150 nm ≫ *d* ≈ 17 nm), *q*(*x*,*y*) images already provide much more details than the corresponding KPFM ones. However, the resolved charge domains are still large (about 200 nm), involving many charges. At this magnification, the distance between two neighbor image points is much larger than the localization length and larger than the correlation length between charges at this temperature (*r*_0_ ≈ 15 nm). The observed net charge at one point weakly interacts with the neighbors, behaving as noninteracting. In fact, for this point size (about 50 × 50 nm^2^) even a single image point may include several charges of the same sign, as its associated size is already larger than the correlation length, which leads to the formation of large domains.^30^

**Figure 2:**
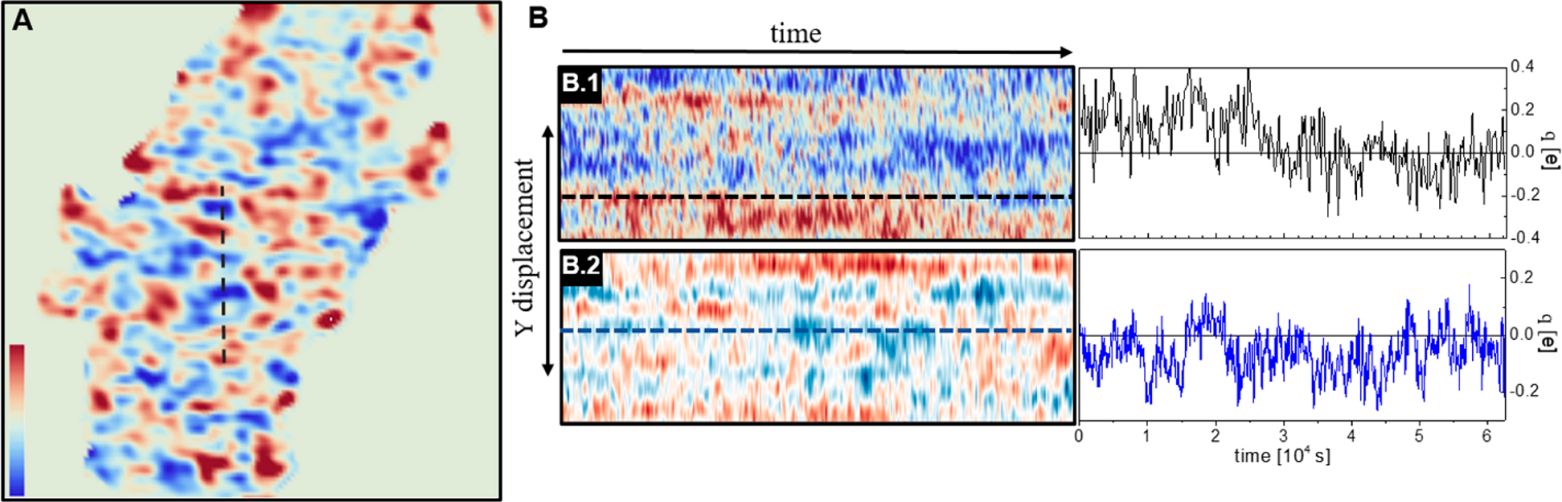
(A) *q*(*x*,*y*) image obtained from a KPFM image (image size 7 × 7 μm). (B) Experimental (B.1) and simulated (arbitrary time units) (B.2) charge domain evolution as a function of time for the vertical section marked in (a) for 292 images (total time 17 h and 20 min 213 s/image) together with a representative line profile in each case.

To study the charge domains dynamics, we acquired successive frames to construct the time evolution movie *q*(*x*,*y*,*t*_i_) (SI Movie 2), and we plot the time evolution of a flake section along all the movie frames (Figure 2B.1). At first glance, we notice that some domains maintain an average charge for a long time (up to several hours) before they change, indicating the existence of very long relaxation times. In addition, looking at the line profile, we also identify charge fluctuation between two successive images around the mean domain charge. This behavior is fully consistent with what we observed in the retrace experiment, although in this case the elapsed time between two measurements at the same point is larger (Δ*t* = 213 s). These fluctuations correspond to charge diffusion at shorter distances than the domain size.

To better understand the charge dynamics over a GO flake, we have simulated the charge distribution and its dynamic with a standard electron glass model together with an efficient kinetic Monte Carlo algorithm.^32,33^ The model considers localization sites distributed randomly in a plane, each site may be empty (*n*_i_ = 0) or occupied (*n*_i_ = 1). The Hamiltonian of the systems is
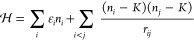
1The first term on the right-hand side contains the site energies, ε_i_ which are considered to be randomly distributed in the interval [−*W*/2,*W*/2], *W* being the strength of disorder. The second term corresponds to the Coulomb interaction between sites, where *K* is the mean occupation of the sites (*K* = 1/2 to ensure charge neutrality). The parameters used for the simulation were *N* = 10 000, a square system with periodic boundary conditions and *L*^2^ = *N* (such that the typical area per site is one), *W* = 2, localization length ξ = 1, and temperature *T* = 0.1, which corresponds to a correlation length of the order of 2.5. We simulate a total of 2 × 10^6^ Monte Carlo steps. To compare experimental and simulated charge densities, we have to coarse-grain the latter applying a Gaussian filter. We use a cutoff radius *r*_c_ = 5,
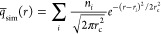
2The results for *q̅*_sim_(*r*) along a line versus time is shown in Figure 2B.2. It is remarkable that even with this simple model, we can qualitatively reproduce both the frame-to-frame fluctuations as well as the long relaxation times confirming our data interpretation.

The existence of very long relaxation times (hours) is further confirmed by analyzing the charge time correlation function
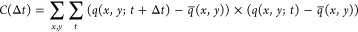
3

In Figure 3, we plot this correlation function on a semilogarithmic scale. We note that *C*(*t*) decays roughly as log(t), indicating that this system presents the characteristic slow relaxation dynamics of electron glasses.^34,35^ Thus, one can then expect to observe other glassy effects in our system, such as memory effects and aging under external excitation.^36^ These effects are usually seen at low temperatures but should also be present at room temperature.^36,37^

**Figure 3:**
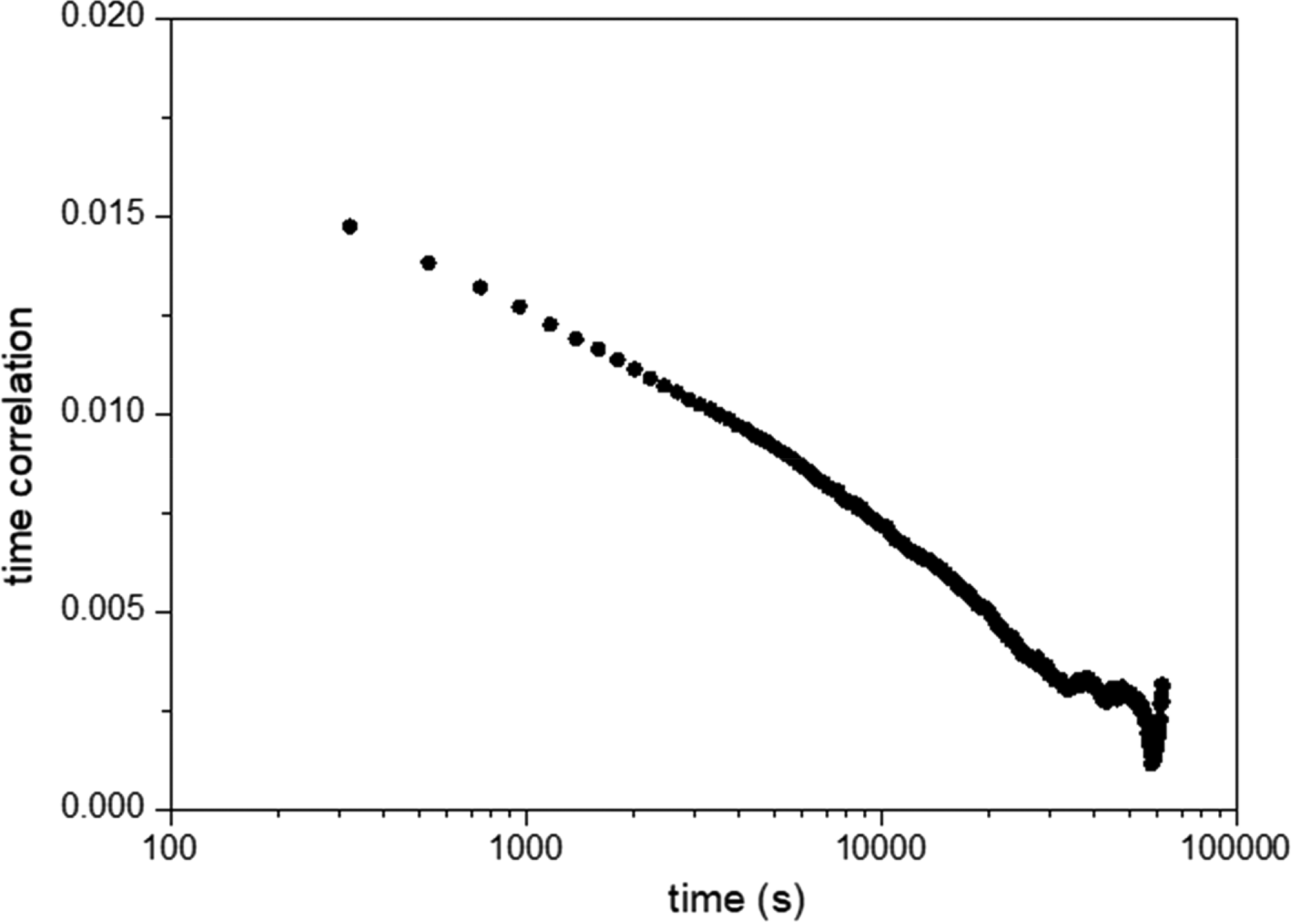
Time correlation function *C*(*t*) calculated from successive charge distribution images *q*(*x*,*y*;*t*) on a semilogarithmic scale obtained from *N* = 292 images (213 s/image).

All this hinders the analysis of the mean and standard deviation (STD) charge images of the movie (Figure SI.6), since extremely long times would be required in order to guarantee convergence. In fact, if we calculate the mean image up to a time *t*_*i*_ (SI Movie 3), its variance on the GO flake region has not converged to a well-defined value (Figure SI.7). Instead, it slowly decays and seems to tend to zero, as we increase *t*. Thus, this supports the idea that there are no regions that tend to trap charges of a particular sign. We also observe that the mean and the standard deviations over time at each image point are not correlated (Figure SI.8). If there were points trapping charges, we would expect, at those points, nonzero mean and small STD. However, we realize that the wrinkles visible in the topography image of the flake show a lower STD signal. This means that the wrinkles in the flake do not attract charges of any particular sign but only reduce the charge mobility.

To get further resolution of the nanoscale charge distribution, we acquire higher-magnification images (*L* = 1.5 μm, *L*/*N* = 12 nm) in a GO flake region (Figure 4). At this magnification, the charge density image (Figure 4C) resolves many individual charge domains of radius about 25 nm. The total charge of the individual domains is around ±1e (Figure 4C). This is fully consistent with the initial estimated charge correlation length (*r*_0_ ≈ 15 nm). For charge distances below *r*_0_, the charge interaction is important and a charge tends to be surrounded by charges of the opposite sign. In these highly oxidized GO (see S2.2) the localization length ξ is below 0.5 nm,^13,15^ while the charge domain radius is of the order of *r*_0_. Thus, these domains will contain many interacting charges but the net domain charge should be close to ±1e. The observed correlation length is consistent with variable-range hopping conduction in the Efros–Shklovskii regime, as an electron jumping a hopping distance (typically 2–6 ξ) still remains in the correlated region where the Coulomb gap should play a role.^38^

**Figure 4:**
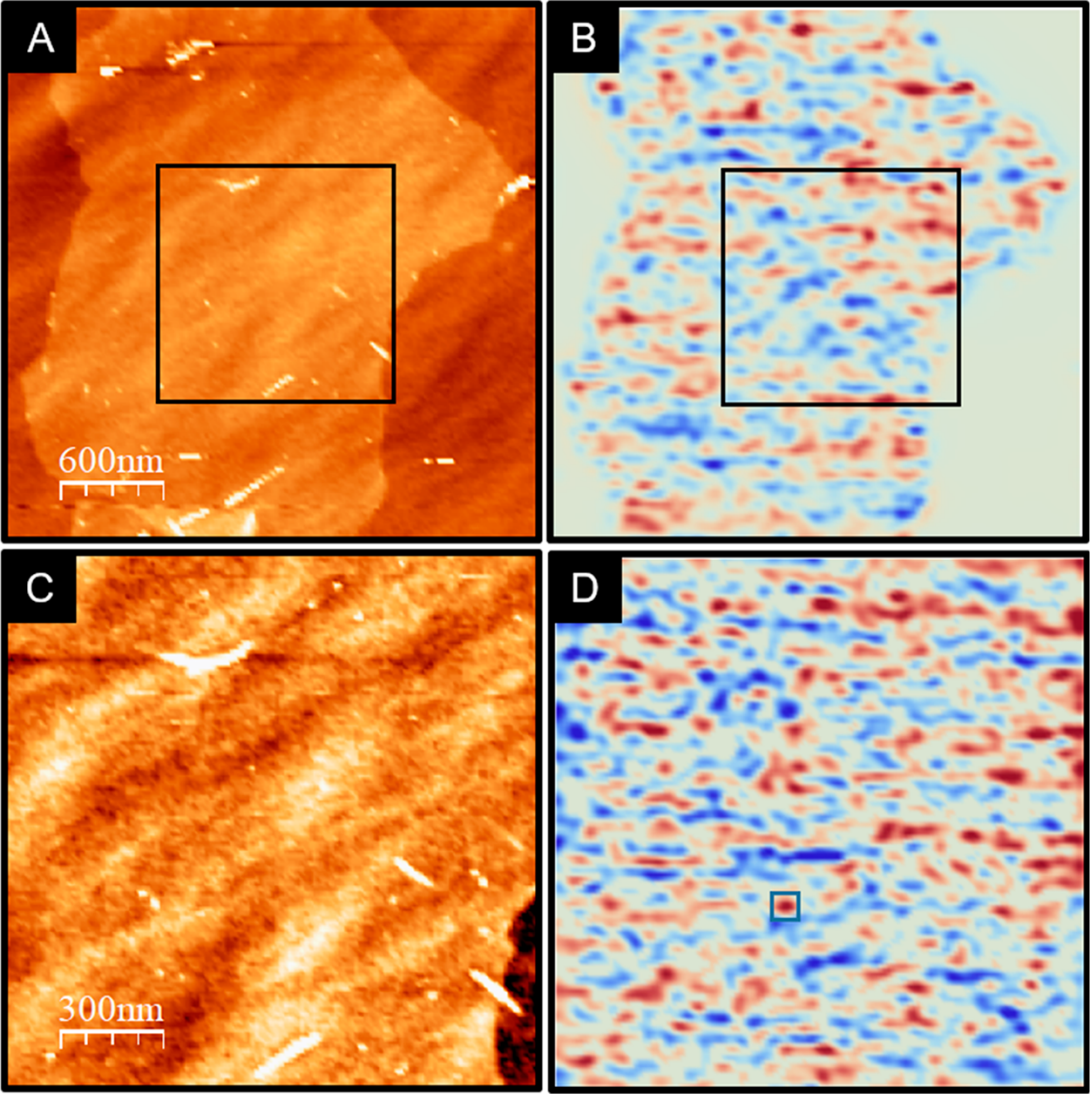
(A) and (C) Topography. (B) and (D) *q*(*x*,*y*) images. In D, the small black square shows a resolved individual domain with total charge + e.

In conclusion, we have been able to measure the nanoscale charge density on GO, showing the existence of localized charge domains. This is a universal feature, because of the large amount of disorder introduced by the functionalization, being verified by the fact that hopping is the main conduction mechanism in GO and r-GO. The charge dynamics shows the existence of long relaxation times (at least hours) and an approximate logarithmic decay of the time correlation function of the charges. These features are typical of electron glasses and therefore indicate that GO could present slow response to some perturbations or even aging and memory phenomena. We emphasize the excellent qualitative agreement of experimental measurements with Monte Carlo simulations confirming the hopping transport.

Moreover, we have reached a lateral resolution up to a scale that allow us to confirm the importance of charge interactions at distances lower than 20 nm. As in highly oxidized GO, this distance is much larger than the localization length, for this system, we can expect Efros–Shklovskii hopping conduction. However, these results could be tuned by modifying the support material as well as varying the oxidation degree to study how it affects the localization and correlation length and therefore the charge dynamics.

In a wider sense, beyond the particular findings on GO, we show that our KPFM methodology is a unique complementary approach to probe the charge transport in 2D disordered systems. In addition, comparison with more complex theoretical models will help to shed light on the unclear transport properties. This new procedure can be further improved using faster scanning speeds, measurements at variable temperature among others.

## Supporting Information Available

Experimental section, two pass method, calculation of the *V*_point_, charge density images from KPFM, mean and STD analysis; complementary spectroscopic studies (XPS, Raman, and FTIR) (PDF)
SI Movie 1: KPFM dynamic as a function of time(AVI)
SI Movie 2: *q*(*x*,*y*) dynamic as a function of time (MP4)
SI Movie 3: Mean image formation as a function of the number of frames (MP4)

## Supplementary Material

tz1c00550_si_001.pdf

tz1c00550_si_002.avi

## References

[ref1] Eda G.; Chhowalla M. Chemically Derived Graphene Oxide: Towards Large-Area Thin-Film Electronics and Optoelectronics. Adv. Mater. 2010, 22, 2392–2415. 10.1002/adma.200903689. 20432408

[ref2] Loh K. P.; Bao Q.; Eda G.; Chhowalla M. Graphene oxide as a chemically tunable platform for optical applications. Nat. Chem. 2010, 2, 1015–1024. 10.1038/nchem.907. 21107364

[ref3] Chen Y.; Lin W.-C.; Liu J.; Dai L. Graphene Oxide-Based Carbon Interconnecting Layer for Polymer Tandem Solar Cells. Nano Lett. 2014, 14, 1467–1471. 10.1021/nl4046284. 24521516

[ref4] Stratakis E.; Savva K.; Konios D.; Petridis C.; Kymakis E. Improving the efficiency of organic photovoltaics by tuning the work function of graphene oxide hole transporting layers. Nanoscale 2014, 6, 6925–6931. 10.1039/C4NR01539H. 24839176

[ref5] Petridis C.; Konios D.; Stylianakis M. M.; Kakavelakis G.; Sygletou M.; Savva K.; Tzourmpakis P.; Krassas M.; Vaenas N.; Stratakis E.; Kymakis E. Solution processed reduced graphene oxide electrodes for organic photovoltaics. Nanoscale Horizon 2016, 1, 375–382. 10.1039/C5NH00089K. 32260627

[ref6] Zibouche N.; Volonakis G.; Giustino F. Graphene Oxide/Perovskite Interfaces for Photovoltaics. J. Phys. Chem. C 2018, 122, 16715–16726. 10.1021/acs.jpcc.8b03230.

[ref7] Jaafar A.; Kemp N. Wavelength dependent light tunable resistive switching graphene oxide nonvolatile memory devices. Carbon 2019, 153, 81–88. 10.1016/j.carbon.2019.07.007.

[ref8] Mkhoyan K. A.; Contryman A. W.; Silcox J.; Stewart D. A.; Eda G.; Mattevi C.; Miller S.; Chhowalla M. Atomic and Electronic Structure of Graphene Oxide. Nano Lett. 2009, 9, 1058–1063. 10.1021/nl8034256. 19199476

[ref9] Mattevi C.; Eda G.; Agnoli S.; Miller S.; Mkhoyan K. A.; Celik O.; Mastrogiovanni D.; Granozzi G.; Garfunkel E.; Chhowalla M. Evolution of Electrical, Chemical and Structural Properties of Transparent and Conducting Chemically Derived Graphene Thin Films. Adv. Funct. Mater. 2009, 19, 2577–2583. 10.1002/adfm.200900166.

[ref10] Gómez-Navarro C.; Meyer J. C.; Sundaram R. S.; Chuvilin A.; Kurasch S.; Burghard M.; Kern K.; Kaiser U. Atomic Structure of Reduced Graphene Oxide. Nano Lett. 2010, 10, 1144–1148. 10.1021/nl9031617. 20199057

[ref11] Tararan A.; Zobelli A.; Benito A. M.; Maser W. K.; Stéphan O. Revisting Graphene Oxide Chemistry via Spatially-Resolved Electron Energy Loss Spectroscopy. Chem. Mater. 2016, 28, 3741–3748. 10.1021/acs.chemmater.6b00590.

[ref12] Eda G.; Mattevi C.; Yamaguchi H.; Kim H.; Chhowalla M. Insulator to Semimetal Transition in Graphene Oxide. J. Phys. Chem. C 2009, 113, 15768–15771. 10.1021/jp9051402.

[ref13] Cheruku R.; Bhaskaram D. S.; Govindaraj G. Variable Range Hopping and Relaxation Mechanism in Graphene Oxide Sheets Containing sp3 Hybridization Induced Localization. J. Mater. Sci.: Mater. Electron. 2018, 29, 9663–9672. 10.1007/s10854-018-9003-6.

[ref14] Joung D.; Khondaker S. I. Efros-Shklovskii Variable-Range Hopping in Reduced Graphene Oxide Sheets of Varying Carbon sp2 Fraction. Phys. Rev. B: Condens. Matter Mater. Phys. 2012, 86, 235423 10.1103/PhysRevB.86.235423.

[ref15] Gómez-Navarro C.; Weitz R. T.; Bittner A. M.; Scolari M.; Mews A.; Burghard M.; Kern K. Electronic Transport Properties of Individual Chemically Reduced Graphene Oxide Sheets. Nano Lett. 2007, 7, 3499–3503. 10.1021/nl072090c. 17944526

[ref16] Kaiser A. B.; Gómez-Navarro C.; Sundaram R.; Burghard M.; Kern K. Electrical Conduction Mechanism in Chemically Derived Graphene Monolayers. Nano Lett. 2009, 9, 1787–1792. 10.1021/nl803698b. 19331348

[ref17] Joung D.; Khondaker S. I. Structural Evolution of Reduced Graphene Oxide of Varying Carbon sp2 Fractions Investigated via Coulomb Blockade Transport. J. Phys. Chem. C 2013, 117, 26776–26782. 10.1021/jp408387b.

[ref18] Debbarma R.; Nguyen N. H. L.; Berry V. Defect Guided Conduction in Graphene-Derivatives and MoS2: Two-Dimensional Nanomaterial Models. Applied Materials Today 2021, 23, 101072 10.1016/j.apmt.2021.101072.

[ref19] Yalcin S. E.; Galande C.; Kappera R.; Yamaguchi H.; Martinez U.; Velizhanin K. A.; Doorn S. K.; Dattelbaum A. M.; Chhowalla M.; Ajayan P. M.; Gupta G.; Mohite A. D. Direct Imaging of Charge Transport in Progressively Reduced Graphene Oxide Using Electrostatic Force Microscopy. ACS Nano 2015, 9, 2981–2988. 10.1021/nn507150q. 25668323

[ref20] Kovtun A.; Candini A.; Vianelli A.; Boschi A.; Dell’Elce S.; Gobbi M.; Kim K. H.; Lara Avila S.; Samorì P.; Affronte M.; Liscio A.; Palermo V. Multiscale Charge Transport in van der Waals Thin Films: Reduced Graphene Oxide as a Case Study. ACS Nano 2021, 15, 2654–2667. 10.1021/acsnano.0c07771. 33464821

[ref21] Yan J.-A.; Chou M. Y. Oxidation Functional Groups on Graphene: Structural and Electronic Properties. Phys. Rev. B: Condens. Matter Mater. Phys. 2010, 82, 125403 10.1103/PhysRevB.82.125403.

[ref22] Jaafar M.; López-Polín G.; Gómez-Navarro C.; Gómez-Herrero J. Step like Surface Potential on Few Layered Graphene Oxide. Appl. Phys. Lett. 2012, 101, 263109 10.1063/1.4773357.

[ref23] Katano S.; Wei T.; Sasajima T.; Kasama R.; Uehara Y. Localized Electronic Structures of Graphene Oxide Studied Using Scanning Tunneling Microscopy and Spectroscopy. Phys. Chem. Chem. Phys. 2018, 20, 17977–17982. 10.1039/C8CP01168K. 29926860

[ref24] Sadewasser S.; Glatzel T. Kelvin Probe Force Microscopy: From Single Charge Detection to Device Characterization; Springer, 2018.

[ref25] Barth C.; Hynninen T.; Bieletzki M.; Henry C. R.; Foster A. S.; Esch F.; Heiz U. AFM Tip Characterization by Kelvin Probe Force Microscopy. New J. Phys. 2010, 12, 093024 10.1088/1367-2630/12/9/093024.

[ref26] Palleau E.; Ressier L.; Borowik Ł.; Mélin T. Numerical Simulations for a Quantitative Analysis of AFM Electrostatic Nanopatterning on PMMA by Kelvin Force Microscopy. Nanotechnology 2010, 21, 225706 10.1088/0957-4484/21/22/225706. 20453285

[ref27] Lacks D. J.; Shinbrot T. Long-Standing and Unresolved Issues in Triboelectric Charging. Nat. Rev. Chem. 2019, 3, 465–476. 10.1038/s41570-019-0115-1.

[ref28] Orihuela M. F.; Somoza A. M.; Colchero J.; Ortuño M.; Palacios-Lidón E. Localized Charge Imaging with Scanning Kelvin Probe Microscopy. Nanotechnology 2017, 28, 025703 10.1088/1361-6528/28/2/025703. 27921998

[ref29] Gonzalez J. F.; Somoza A. M.; Palacios-Lidón E. Charge Distribution from SKPM Images. Phys. Chem. Chem. Phys. 2017, 19, 27299–27304. 10.1039/C7CP05401G. 28967652

[ref30] Somoza A. M.; Palacios-Lidón E. Localized Charges in Thin Films by Kelvin Probe Force Microscopy: From Single to Multiple Charges. Phys. Rev. B: Condens. Matter Mater. Phys. 2020, 101, 075432 10.1103/PhysRevB.101.075432.

[ref31] Escasaín E.; López-Elvira E.; Baró A. M.; Colchero J.; Palacios-Lidón E. Nanoscale Electro-Optical Properties of Organic Semiconducting Thin Films: From Individual Materials to the Blend. J. Phys. Chem. C 2012, 116, 17919–17927. 10.1021/jp304278w.

[ref32] Pollak M.; Ortuno M.; Frydman A. The Electron Glass; Cambridge University Press: New York, 2013.

[ref33] Tsigankov D. N.; Efros A. L. Variable Range Hopping in Two-Dimensional Systems of Interacting Electrons. Phys. Rev. Lett. 2002, 88, 176602 10.1103/PhysRevLett.88.176602. 12005771

[ref34] Ovadyahu Z. Relaxation dynamics in quantum electron-glasses. Phys. Rev. Lett. 2007, 99, 226603 10.1103/PhysRevLett.99.226603. 18233309

[ref35] Ovadyahu Z. Slow Dynamics of the Electron-Glasses; the Role of Disorder. Phys. Rev. B: Condens. Matter Mater. Phys. 2017, 95, 134203 10.1103/PhysRevB.95.134203.

[ref36] Grenet T.; Delahaye J. Manifestation of ageing in the low temperature conductance of disordered insulators. Eur. Phys. J. B 2010, 76, 229 10.1140/epjb/e2010-00171-9.

[ref37] Ortuño M.; Escasain E.; Lopez-Elvira E.; Somoza A. M.; Colchero J.; Palacios-Lidon E. Conducting Polymers as Electron Glasses: Surface Charge Domains and Slow Relaxation. Sci. Rep. 2016, 6, 21647 10.1038/srep21647. 26911652 PMC4766496

[ref38] Efros A. L.; Shklovskii B. I. Coulomb gap and low-temperature conductivity of disordered systems. J. Phys. C: Solid State Phys. 1975, 8, L49 10.1088/0022-3719/8/4/003.

