# Supplementary material for: Nanoscale Charge Density and Dynamics in Graphene Oxide
Source: ACS Mater Lett. 2021 Nov 22;3(12):1826–31. doi: 10.1021/acsmaterialslett.1c00550 (PMC8655804; doi:10.1021/acsmaterialslett.1c00550)
Supplement: Supplementary file 1 — tz1c00550_si_001.pdf [file tz1c00550_si_001.pdf]

Nanoscale Charge Density and Dynamics in Graphene Oxide

Elisa Palacios-Lidón,† Jaime Colchero,† Miguel Ortuno, † Eduardo Colom,‡ Ana M. Benito,‡ Wolfgang K. Maser,‡ Andrés M. Somoza*†*

†Departamento Física, Edificio CIOyN (Campus Espinardo), Universidad de Murcia, E-30100 Murcia, Spain

‡Instituto de Carboquímica (ICB-CSIC), E-500018, Spain

SI.1. Experimental

In low conducting very thin materials such as GO, the intensity of the localized charge contribution to the V_{KPFM} signal increases as the relative permittivity of underlying substrate decreases [1]. For that reason, we have used a thick 300nm SiO_2 ($\varepsilon_r \approx 4.3$) on Si. In addition, all the measurements have been carried on under a dry nitrogen atmosphere to avoid water-screening effects. To highlight the influence of these two parameters, in **Figure SI.1** we show the KPFM images of a GO flake deposited between two gold stripes evaporated on a 300 nm SiO_2/Si substrate at different relative humidity (RH). We note that the charge domains are detected only at low humidity and only on the SiO_2 channel. In this case, the V_{charge} contribution is large enough to be detected and superimposed on the $V_{CP} \approx 80$mV contribution (that is essentially an offset). On the contrary, charge domains cannot be resolved on the Au substrate even at low humidity. Due to the high Au permittivity, the V_{charge} contribution is so

low, that GO flakes on Au shows a constant V_{KPFM}, that correspond only to the V_{CP} contribution.

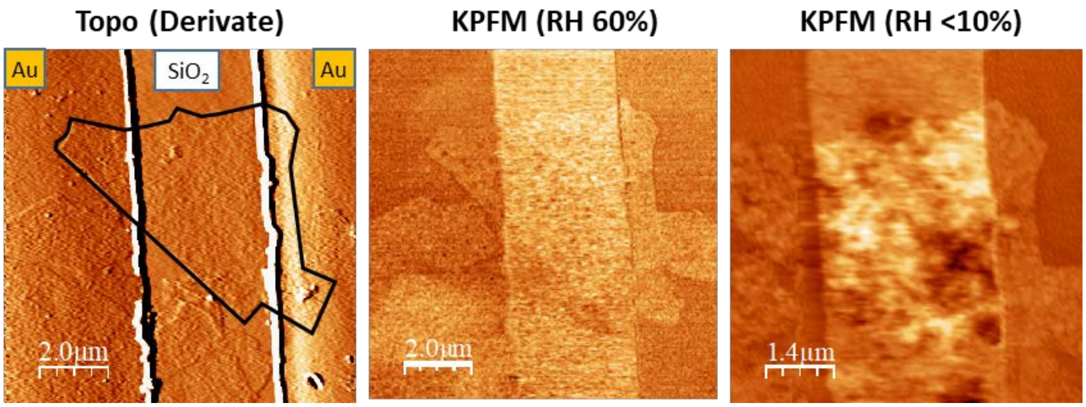

Figure SI.1. Individual GO flake deposited between two Au stripes. KPFM images acquired at different RH (KPFM z scale=400 mV)

SI.2. Two-pass measurements

In the two-pass acquisition mode, the tip scans each fast scan line twice before it moves to the next scanning line. In this way, two simultaneous images are obtained. Since the two lines are acquired very close in time, they can be directly compared neglecting artefacts due to drift, tip changes, etc. Typically, this mode is used to study the influence of one or several parameters on the measurements by changing them between the first and the second pass (tip-sample distance, illumination, oscillation amplitude, etc). Since our goal is to study the charge dynamics, in this work the same acquisition parameters (set-point, scan speed and feedback parameters) are used. **Figure SI.2** shows the first and second pass images together with the line by line subtracted image. We clearly see that on the GO flake there is a larger fluctuation than on the SiO_2 substrate. A line profile shows that these fluctuations are no larger than ±60mV but well above the substrate noise level σ_{noise}≈10mV.

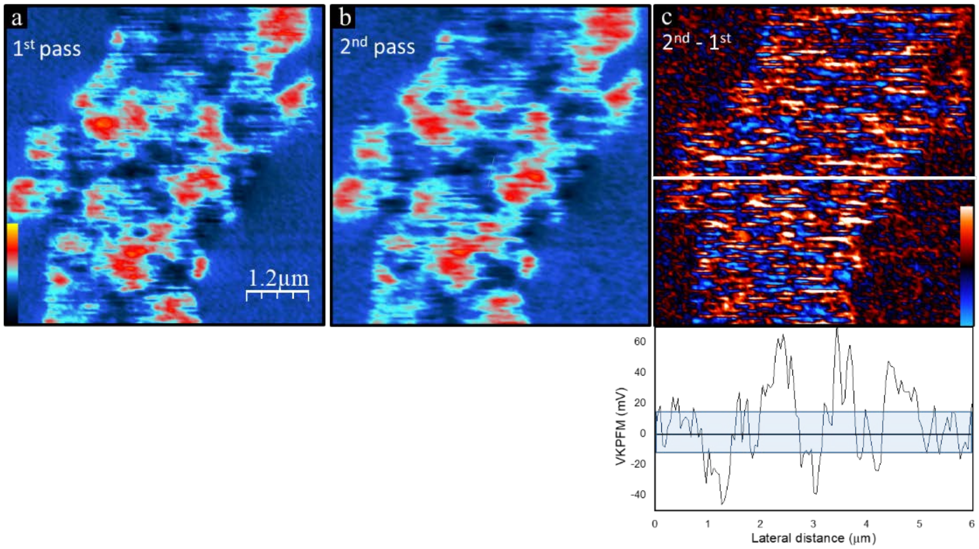

Figure SI.2. (a) and (b) First and second pass KPFM images respectively (z scale 400mV), **(c)** subtracted image (z scale = ± 60mV) together with a line profil underlining the intensity fluctuations. The grey rectangle indicates the substrate noise level ($\sigma_{noise} \approx 10mV$).

To estimate how much charge has moved between two pass lines to produce such V_{charge} change, we need to calculate the V_{charge} signal that would produce one charge (V_{point}) in a GO flake deposited on a SiO_2 substrate, and how it changes with the lateral distance.

SI.3. Calculation of the V_{point}

To obtain the V_{point} signal (**Fig. SI.3**) that would produce a single point charge on a monolayer of GO, we have used the image charge-based method proposed in ref [1]. This V_{point}, allows us to estimate the effective amount of charge that has moved at a point within the time scale of the two pass (2s in our case) to produce the observed change in V_{KPFM} (less than ±60mV). We find that it corresponds to a charge redistribution in which effectively an electron has travel a distance no larger than 10 nm.

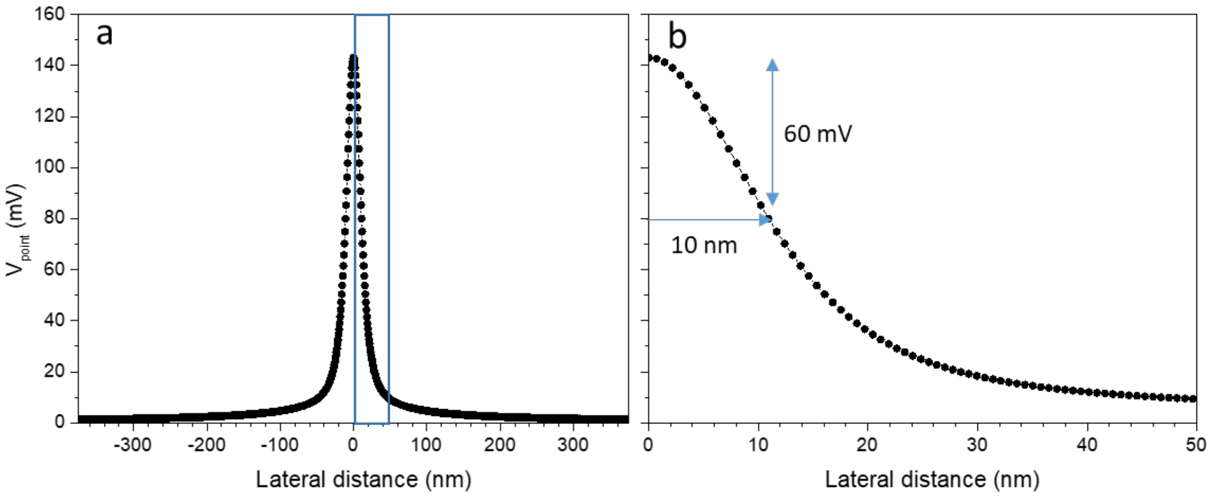

Figure SI.3. (a) V_{point} generated by a point charge on a GO monolayer. **(b)** Zoom of the blue rectangular region in (a).

For the calculation, we have used a tip radius R=15 nm, tip sample distance d=7 nm and a SiO_2 relative permittivity ε_{SiO2}=4.3. As explained above, these parameters are obtained as proposed in ref.[**¡Error! Marcador no definido.**]. In addition, we have used a GO thickness h=1.5 nm (obtained from topography images) and ε_{GO}=4.3, similar to the one of SiO_2. The ε_{GO} of a single GO flake is not precisely known. From our measurements, we see no difference between the SiO_2 and the GO flakes in the capacitance images obtained from the $2\omega_{elec,}$ signal while they show a clearly lower permittivity than silicon if the GO is deposited directly on it (not shown). We estimate that in the perpendicular direction, the ε_{GO} should be between 3.5 and6. Variation on the ε_{GO} in our calculations between these values results in a charge density variation less than 10%, since for a very thin film layer, the V_{point} is mainly determined by the ε_r of the substrate.

SI. 4. Charge density image from the V_{KPFM} image.

To calculate the charge density image q(x,y) we use Fast Fourier Transform based algorithms (FFT) described in ref [2]. To do so, we first calculate the V_{point} image as explained above and we use it for the deconvolution of the V_{KPFM} images (**Fig.SI. 4**). Due to experimental noise, in the deconvolution process a circular Gaussian filter in k-space to remove non-physical artifacts is applied. In this work, we have selected the corresponding filter radius (k_c) as such that $\sigma_{noise}(k_c)/V_{point}(k_c) \approx 1$, as explained in ref [1]. The use of this filter limits the lateral resolution broadening the charge peaks and blurring the details smaller than a radius $R_c=2\pi/k_c$.

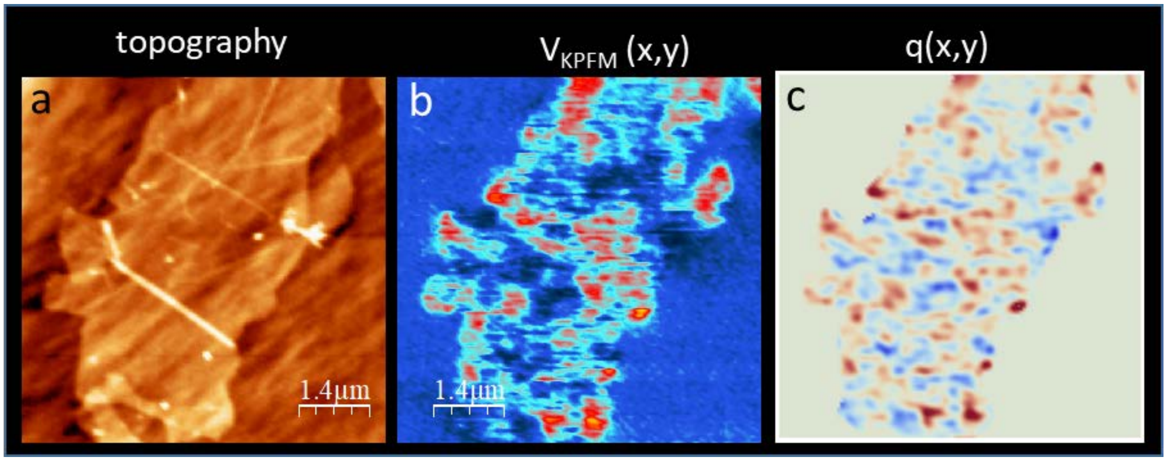

Figure SI.4. (a) Topography (Δz=4nm) and **(b)** V_{KPFM} (Δz=400 mV) images of an individual single layer GO flake. **(c)** Corresponding q(x,y) image obtained from (b) with the deconvolution method.

In **Figure SI.5** we have added to the ideal noise-free $V_{point}(x,y)$ image a random Gaussian noise (σ=10 mV) and we have obtained the corresponding $q_{point}(x,y)$ image using exactly the same k_c filter radius used in our experimental $V_{KPFM}(x,y)$ images. In this way, we can estimate the charge distribution produced by one punctual electron in our system including

the experimental noise. We notice that although the peak is broad (due to the filter), the charge is conserved under the integration of the peak.

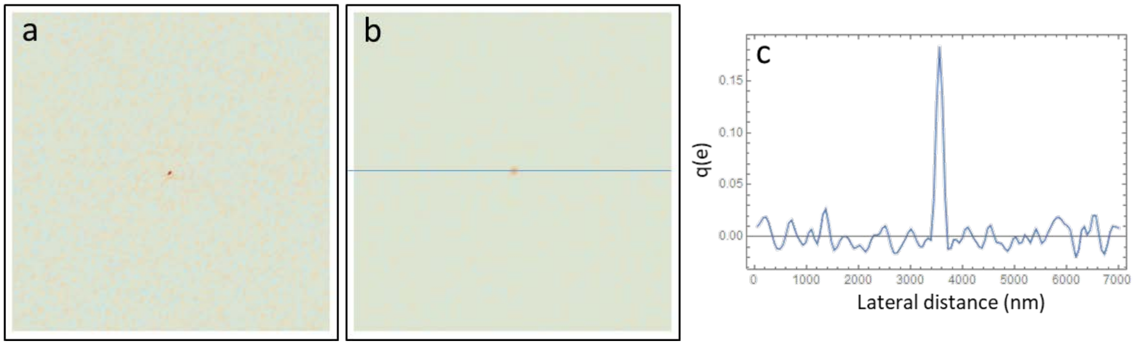

Figure SI.5. (a) KPFM image generated by a point charge (V_{point} image) with an additional Gaussian random noise σ_{noise}≈10mV. **(b)** Corresponding q(x,y) image. **(c)** Line profile marked in (b).

SI.5 Mean and STD images from the movie.

We have calculated the mean and the STD images

$$\bar{q}(x,y) = \frac{1}{N}\sum_i q(x,y;t_i) \quad (1)$$

$$\sigma(x,y) = \sqrt{\frac{1}{N}\sum_i q^2(x,y;t_i) - \bar{q}^2(x,y)} \quad (2)$$

of a charge density movie ($q(x,y,t_i)$) of N=292 frames (total time 17h and 20 min and 213s/frame) shown in **Figure SI. 6**.

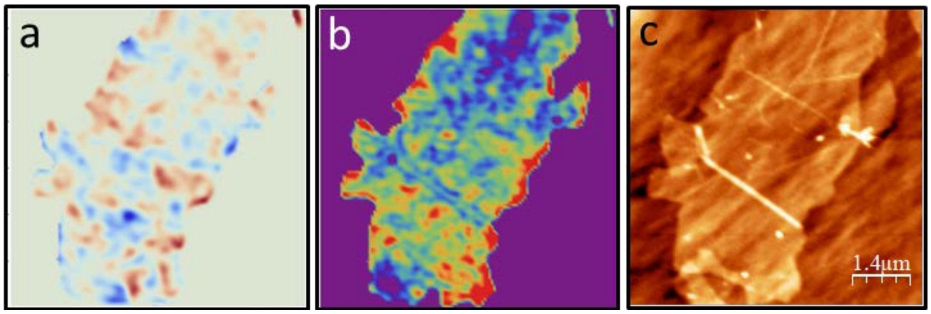

Figure SI.6. (a) Mean **(b)** STD and **(c)** mean topography obtained from the q(x,y) movie for a total of N=292 frames.

We have also calculated the mean image as a function of the number of accumulated frames n (n=1,….,N) (**SImovie_3**).

$$\bar{q}(x,y,n) = \sum_{i=1}^{n} q(x,y,i)/n \qquad (3)$$

Applying a mask analysis, we calculate the variance of the mean frame ($\sigma_{\bar{q}}^2(n)$) of the GO flake as a function of number of averaged frames.

$$\sigma_{\bar{q}}^2(n) = \frac{1}{N}\sum_{x_{flake}}\sum_{y_{flake}}(\bar{q}(x,y,n) - \bar{q}(n))^2 \qquad (4)$$

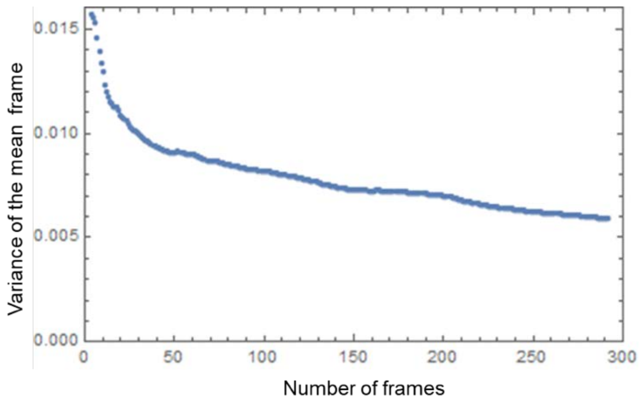

Figure SI.7. Variance of $\bar{q}(x,y,n)$ as a function of the number n of averaged frames.

We see in **Figure. SI. 7** that it has not converged yet to a well-defined value. Instead, it slowly decreased. Thus, we could expect that even the domains that are still are appreciated in **Figure.SI 6(a)** could probably disappear if the observation time is much longer.

The STD image (**Fig SI.6 (b)**) presents different regions with large and small STD, but the mean and the STD images are very weakly correlated (**Fig. SI. 8)**, except at the flake border where due to the applied mask the procedure is not fully reliable.

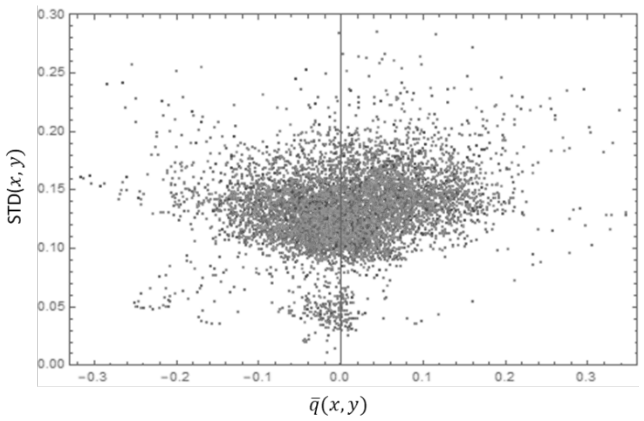

Figure SI.8 Dispersion diagram of $\{\bar{q}(x, y, n), STD(x, y, n)\}$ from images **SI.6 (a)** and **(b)**.

We have analyzed the origin of such STD lateral structure finding that it is mainly due to the long-time fluctuations; while the short-time fluctuations are essentially homogeneous over the flake This fact confirms our expectation that there are no fixed net charges in the flake. If a region has a large probability to keep a charge of a given sign, the STD in that region should be smaller. However, we realize that the flake twists visible in the topography shows a lower STD signal. This means that the twists in the flake do not attract charges of any particular sign, but reduce the charge mobility.

SI.6 Complementary Characterization

Complementary spectroscopic studies were performed on powder samples of GO obtained by freeze-drying the purchased GO dispersion.

X-Ray Photoelectron Spectroscopy (XPS)

XPS analysis of GO powder was carried out using an ESCA Plus Omicron spectrometer using a monochromatized Mg X-ray source (1253.6 eV). Surface charging effects due to the

insulating nature of GO needed to be compensated by a shift of the XPS spectrum by 3.3 eV. A Shirley background was subtracted and the XPS peaks were fitted with a GL(70) function, whose line-shape accounts for 70% Gaussian and 30% Lorentzian character. The full width half maximum (FWHM) values were fixed to a maximum of 1.6 eV. Asymmetry for the sp^2 carbon peak was defined by a line-shape asymmetry function LF (1, 2, 57, 0). Figure S2.1. shows the C1s spectra of GO deconvoluted in 5 components located at 284.1 eV (C=C bonds), 285.0 eV (C–C bonds), 287.0 eV (C–O bonds) and 287.6 eV (C=O) and 288.6 eV (COOH). The atomic contribution of the respective components amounts to 5.2 at% (C=C), 40.7 at% (C–C), 34.0 at% (C–O), 13.6 at% (C=O) and 6.4 at% (COOH). The elemental composition according to the survey spectra reveals a C/O ratio of 2.7.

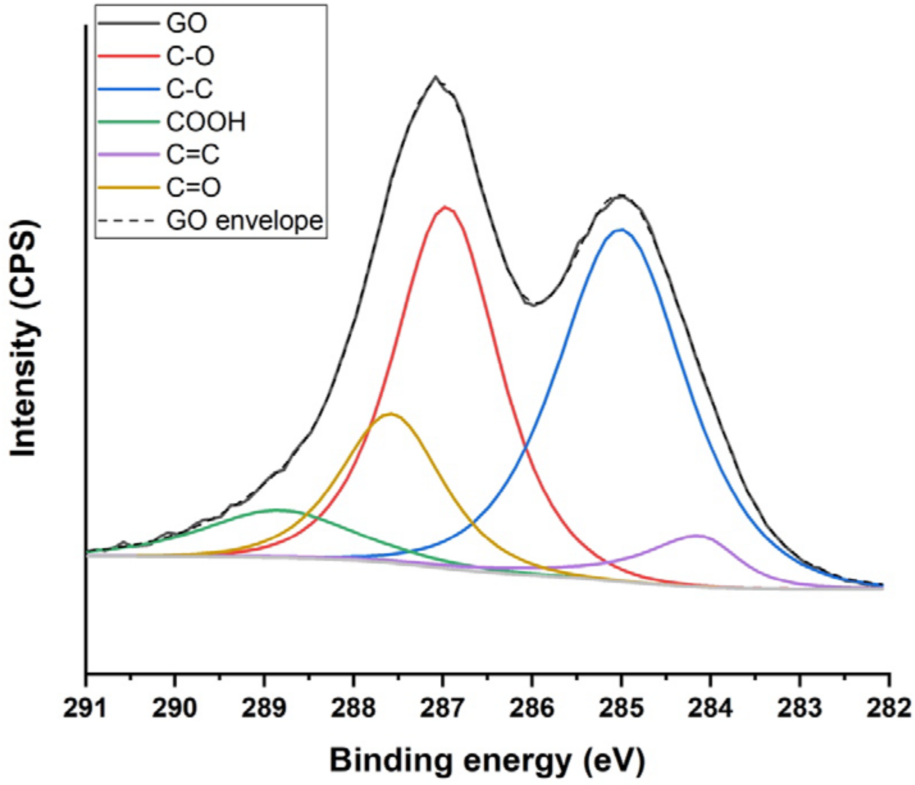

Figure SI.9. XPS C1s spectra of GO and fitted envelope with the deconvoluted components.

Raman Spectroscopy

Raman spectroscopy on GO powder deposited on a glass substrate was performed using a Horiba Jobin-Yvon HRLAB HR 800 spectrometer at a laser excitation length of 514 nm on GO. The Raman spectrum of GO (Figure S.2.2) clearly exhibits the characteristic G band at 1580 cm^{-1}, indicative for in-plane vibrations from carbon atoms in a sp^2 bonded carbon lattice, and a strong D band at about 1350 cm^{-1}, attributed to defects and disordered carbon atoms in the carbon lattice, suggesting a high number of structural imperfections, most likely due to the presence of oxygen groups on the basal plane of the carbon lattice and other defects induced during the harsh oxidative conditions under which GO typically is prepared. The G band appears to be broadened. This is typical for highly defective structures and results in the presence of the Raman active D' band at about 1610 cm^{-1}. The deconvolution of the spectra requires a further component to account for the rather high background between the D and the G band. This component is designed as D* peak and observed in highly defective samples [6] Its origin is under discussion but eventually may be related to finite size of graphitic crystallites. With this four contributions, the Raman spectrum could be successfully deconvoluted, resulting in peak positions for the D', G, D* and D band at 1608 cm^{-1}, 1580 cm^{-1}, 1503 cm^{-1} and 1353 cm^{-1}, respectively, and an intensity ratio of the D and G band with a value of $I_D/I_G = 1.28$. From this value the crystallite size of the sp^2 domains can be estimated applying the following empirical equation, according to the studies of M.A. Pimenta [7,8]:

$$L_a = 2.4 \cdot 10^{-10} \cdot \lambda^4_{laser} \cdot (I_D/I_G)^{-1} \quad (5)$$

Here, λ_{laser} corresponds to the Raman excitation wavelength in nm and I_D/I_G to the Intensity ratio of the D/G line. Excited at 514 nm and taking the resulting I_D/I_G value of 1.28, a crystallite size of the sp^2 domains in GO of about 13 nm is obtained.

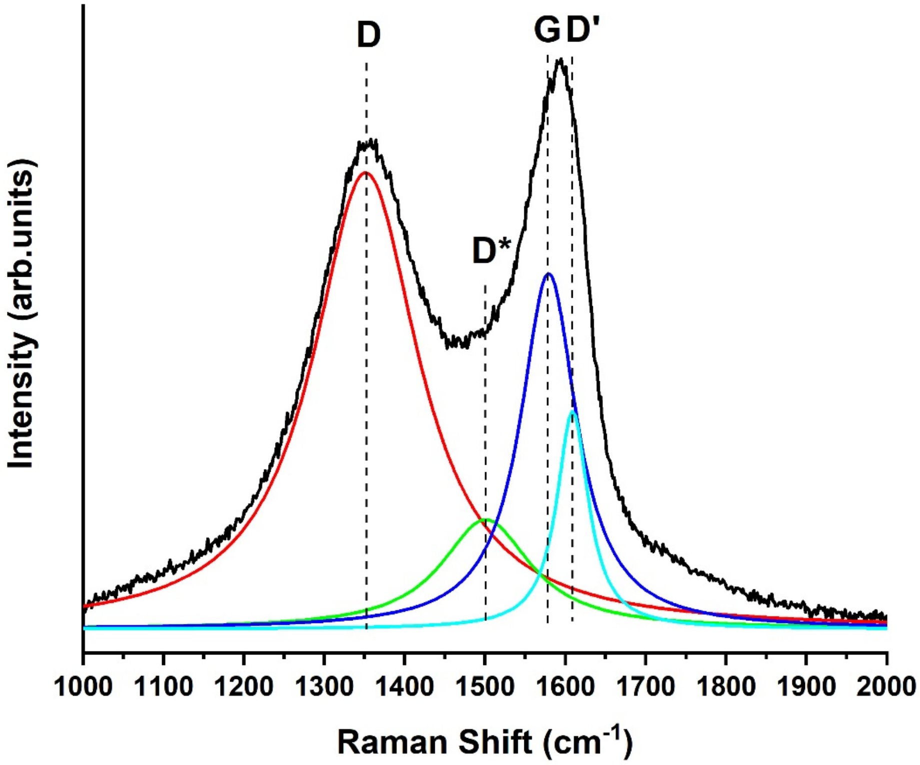

Figure SI.10. Raman spectrum of GO and the deconvoluted D, D*, G and D' band.

Fourier Transform Infrared (FTIR) Spectroscopy:

FTIR analysis of GO powder in KBr pressed pellet was performed using a Bruker Vertex 70 spectrometer. The spectrum (Figure S2.3) reveals the presence of various types of oxygen functional groups. An intense band in the region of 3000 – 3600 cm^{-1} characteristic of stretching vibrations of O–H groups, originates from alcohols, carboxylic acids and water. The peak at 1735 cm^{-1} can be assigned to C=O stretching modes, compatible with the

presence of ester and lactone groups in GO. The peak at 1625 cm^{-1} corresponds to the scissor mode of water (deformation vibration), which overlaps the asymmetric stretch mode of sp^2 C=C vibrations commonly appearing at 1580-1600 cm^{-1}. The vibrations at 1230 cm^{-1} and 1050 cm^{-1} can be ascribed to alcohol (C–OH) and epoxy (C–O-C) groups, respectively.

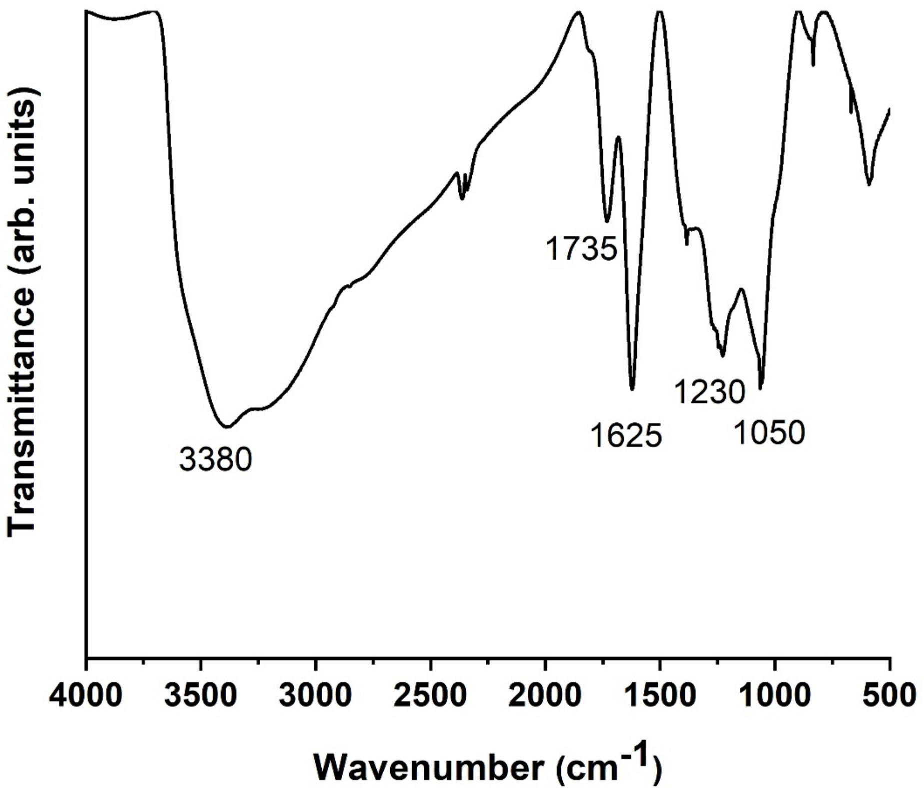

Figure SI.11. FTIR spectrum of GO

[1] Somoza, A. M.; Palacios-Lidón, E. Localized Charges in Thin Films by Kelvin Probe Force Microscopy: From Single to Multiple Charges. *Phys. Rev. B* **2020**, *101* (7), 075432.

[2] Gonzalez, J. F.; Somoza, A. M.; Palacios-Lidón, E. Charge Distribution from SKPM Images. *Phys. Chem. Chem. Phys.* **2017**, *19* (40), 27299–27304.

[6] Kaniyoor, A.; Ramaprabhu, S. A Raman spectroscopic investigation of graphite oxide derived graphene. *AIP Advances* **2012**, 2, 032183.
[7] Pimenta, M. A.; Dresselhaus, G.; Dresselhaus, M. S.; Cançado, L. G.; Jorio, A.; Saito, R. Studying disorder in graphite-based systmes by Raman spectroscopy. *Phys. Chem. Chem. Phys.* **2007**, 9, 1276-1291
[8] Cançado, L.G.; Takai, K.; Enoki, T.; Endo, M.; Kim, Y. A.; Mizusaki, H.; Jorio, A.; Coelho, L.N. Magalhães-Paniago, R.; Pimenta, M. A. General Equation for the Determination of the Crystallite Size La of Nanographite by Raman Spectroscopy. *Appl. Phys. Lett.* **2006**, 88, 1-4.
